# The Expression and Distributions of ANP32A in the Developing Brain

**DOI:** 10.1155/2015/207347

**Published:** 2015-03-19

**Authors:** Shanshan Wang, Yunliang Wang, Qingshan Lu, Xinshan Liu, Fuyu Wang, Xiaodong Ma, Chunping Cui, Chenghe Shi, Jinfeng Li, Dajin Zhang

**Affiliations:** ^1^Weifang Medical University, Weifang 261042, China; ^2^Department of Neurology, The 148th Hospital, Zibo 255300, China; ^3^Zhengzhou University, Zhengzhou 450001, China; ^4^Department of Neurosurgery, PLA 301 Hospital, Beijing 100853, China; ^5^Center for Basic Medical Sciences, Navy General Hospital of Chinese PLA, Beijing 100048, China

## Abstract

Acidic (leucine-rich) nuclear phosphoprotein 32 family, member A (ANP32A), has multiple functions involved in neuritogenesis, transcriptional regulation, and apoptosis. However, whether ANP32A has an effect on the mammalian developing brain is still in question. In this study, it was shown that brain was the organ that expressed the most abundant ANP32A by human multiple tissue expression (MTE) array. The distribution of ANP32A in the different adult brain areas was diverse dramatically, with high expression in cerebellum, temporal lobe, and cerebral cortex and with low expression in pons, medulla oblongata, and spinal cord. The expression of ANP32A was higher in the adult brain than in the fetal brain of not only humans but also mice in a time-dependent manner. ANP32A signals were dispersed accordantly in embryonic mouse brain. However, ANP32A was abundant in the granular layer of the cerebellum and the cerebral cortex when the mice were growing up, as well as in the Purkinje cells of the cerebellum. The variation of expression levels and distribution of ANP32A in the developing brain would imply that ANP32A may play an important role in mammalian brain development, especially in the differentiation and function of neurons in the cerebellum and the cerebral cortex.

## 1. Introduction

ANP32A is a member of acidic nuclear phosphoprotein 32 kDa (ANP32) family [1, 2]. The nomenclature of ANP32A family members is confusing because the same protein has been given more than one name based on the context of isolation. The family members are comprised of ANP32A (also known as PP32, LANP, HPPCn, I1PP2A, MAPM, or PHAPIa), ANP32B (PAL31, APRIL, or PHAPIb) [3], ANP32C (PP32r1) [4], ANP32D (PP32r2) [5], and ANP32E (Cpd1, LANP-L, or PHAPIII) [6].

All ANP32 proteins share two highly conserved regions: the N-terminal leucine-rich repeats (LRRs) sequence and the C-terminal acidic tail [7]. For ANP32A, the hydrophobic LRRs shape a globular head domain. LRRs, belonging to a superfamily with diverse bioactivity, may potentially function in mediating protein-protein interaction [8]. The extended and hydrophilic C-terminal domain is highly unusual in its amino acid composition, containing abundant aspartic and glutamic acid residues, and a putative nuclear localization signal (NLS) [9]. According to the structure characteristic, it is not surprising to find ANP32 proteins involved in a variety of cellular processes in both nucleus and cytoplasm, including signaling, apoptosis, protein degradation, and morphogenesis.

More studies have focused on ANP32A, the founding member of the ANP32 family. ANP32A (PP32) was originally found as a tumor suppressor [10], associated with cancer cell survival and drug efficacy. Meanwhile its closely related homologue PP32r1 is oncogenic and is overexpressed in breast cancer and prostate cancer [11]. Family member ANP32B was indicated as a potential prognostic marker of human breast cancer [2]. In fact, ANP32A has also been identified having a potential oncogenic and drug-resistant function in hepatocellular carcinoma [12], colorectal cancer [13], and pancreatic tumor [14]. The confused role in tumorigenesis of ANP32A may be due to its functions and subcellular localizations being almost bewildering in variety.

ANP32A is known to be a key component of the inhibitor of acetyltransferase (INHAT) complex in the nucleus, involved in regulating chromatin remodeling or transcription initiation [15]. ANP32A forms a multisubunit heterocomplex with HuR, regulating the nucleocytoplasmic shuttling of HuR, which is essential for RNA stability and transport [16]. In the cytoplasm, ANP32A (mapmodulin) is positioned to microtubule associated proteins (MAPs), involved in regulating microtubule function and microtubule-based vesicular trafficking [17]. ANP32A may control enzymatic activities by inhibition of protein phosphatase 2A (PP2A) or activation of caspases [18]. ANP32A (LANP) and SET were observed at the inner surface of the plasma membrane of lymphocytes and supposedly play a role in signal transduction [19]. ANP32A (HPPCn) was the first factor that it could transport to the extracellular space and act as an autocrine factor to promote DNA synthesis and suppress apoptosis by upregulating myeloid cell leukemia-1 [20]. These functions taken together suggest that ANP32A could therefore be an important regulator of cellular homeostasis.

ANP32A plays essential roles in a variety of neural pathophysiology processes. The level of ANP32A (I1PP2A) is increased in Alzheimer's disease (AD) and may be involved in regulatory mechanism of affecting Tau phosphorylation and impairing the microtubule network and neurite outgrowth [21]. ANP32A (LANP) regulates neuronal differentiation by epigenetic modulating expression of the neurofilament light chain, an important neuron-specific cytoskeletal gene [22]. ANP32A can interact with the retinoblastoma protein Rb in both young and mature neurons and is implicated in the regulation of neuronal survival by CXCL12/CXCR4 [23]. The decreased levels of ANP32A, as a potent and selective PP2A inhibitor, may contribute to abnormal neuritic morphology in a dominantly inherited neurodegenerative disorder of the spinocerebellar ataxia type 1 [24].

The expression characteristic of ANP32A in the developing brain, especially details on the expression and distributions of ANP32A in the human brain, had rarely been reported [25]. In this study, the distribution of ANP32A in different human brain areas, as well as ANP32A abundance in the human fetal and adult brain, was identified. For more details of the expression and localization of ANP32A in the developing brain, a series of different time point mouse brains from embryonic stage to adult stage was harvested and analyzed with ANP32A specific primers and antibodies. To explore ANP32A in the development of the nervous system, it could provide crucial information about pivotal roles of the protein in morphogenetic process and regulating mechanisms.

## 2. Materials and Methods

### 2.1. Animals

Adult C57 BL/6 mice were kept with free accesses to food and water. The day of insemination was designated as embryonic day 0 (E0). The day of birth was designated as postnatal day 0 (P0). Brains from different embryonic period (E12 and E16), early time points after birth (P0, P5, and P12), pubescent male mice (approximately 5-6 weeks old), and adult male mice (approximately 8–10 weeks old) were frozen quickly and stored at −80°C until required for experiments. E12 and E16 brains were collected under the dissection microscope and the mesenchymal tissues were removed with fine forceps as much as possible. For immunohistochemistry preparation, brains were fixed in formalin (4% formaldehyde in 1× PBS, pH7.4) for 24 hours and then embedded in paraffin. Serial sections (4 *μ*m) were mounted onto silane-coated slides (Dako, Denmark).

### 2.2. Human Multiple Tissue Expression (MTE) Array Analysis

The human MTE array (BD, America) was a positively charged nylon membrane to which poly A^+^ RNAs from different human tissues had been normalized and immobilized in separate dots, along with several controls. The MTE array made it possible to determine the relative expression levels of a target mRNA in different tissues and developmental stages [26]. To this end, poly A^+^ RNA samples on each MTE array had been normalized to the mRNA expression levels of eight different “housekeeping” genes, which minimized the small tissue-specific variations in expression of any single housekeeping gene [27]. The human MTE array gave a relative convenient and convincing way to demonstrate the levels of ANP32A mRNA in 20 different areas of the human brain, as well as adult whole brain and fetal brain.

A 750 bp* ANP32A* probe was amplified from human ANP32A gene (Gene ID: 8125) in the pET-24a(+) plasmid (kept by our laboratory) with primers 5′-CGGGATCCATGGAGATGGGCAGACGGATT-3′ (forward) and 5′-AACTGCAGGTCAT-CATCTTCTCCCTCATC-3′ (reverse). Probe used for the human MTE assay was radioactively labeled with *α*-[^32^P]dCTP using the DNA labeling kit (Promega, America) as described by the operation manual. Prehybridization of the MTE array was performed at 68°C for 2 hours in ExpressHyb hybridization (Clontech, America) with sheared salmon testis DNA added to a final concentration of 0.1 mg/mL. The denatured radiolabeled probe was mixed directly into the prehybridization solution and hybridized overnight at 68°C. After hybridization, the array was washed three times at 37°C in solution 2× SSC with 0.1% SDS for 10 min each time, repeated at 65°C in solution 0.1× SSC with 0.1% SDS for 20 min each time. Array was exposed to X-ray film at −70°C for 48 hours. ANP32A mRNA levels were determined by densitometric scanning of autoradiographs.

### 2.3. Western Blotting

Mouse whole brain tissues were homogenized in RIPA buffer (Sigma, America) on ice, lysates were centrifuged at 12,000 ×g, 4°C for 15 minutes, and supernatants were collected for western blot analysis. Protein concentration of samples was determined by the Bio-Rad protein Assay (Bio-Rad, America). Protein extracts (20 *μ*g) were fractionated by 12% SDS-PAGE and then transferred to PVDF membranes by the Trans-Blot semidry transfer cell (Bio-Rad, America). Membranes were subsequently blocked with 10% skim milk in 1× PBS and then incubated with a rabbit polyclonal antibody against ANP32A (1 *μ*g/mL, ab 5991, dilution 1 : 1000) (Abcam, British) or *β*-actin (loading control, number 4970, dilution 1 : 1000) (CST, America). Following washing with PBST buffer, membranes were incubated with secondary antibody at appropriate dilution in 5 mL 10% blocking buffer. Protein bands on blots were detected by enhanced chemiluminescence (Applygen Technologies Inc., China) and visualized by LAS-4000 (GE, America).

### 2.4. Quantitative Real-Time RT-PCR

Total RNA was extracted from mouse whole brains using a Trizol (Invitrogen, America). 1 *μ*g of total RNA was converted to cDNA using FastQuant RT kit (with gDNase) (TIANGEN BIOTECH, China). cDNA samples were then used as templates for quantitative real-time PCR by the SuperReal Premix Plus (SYBR Green) (TIANGEN BIOTECH, China) with a Bio-Rad Chromo 4 real-time PCR detector (Bio-Rad, CA, USA). The mouse ANP32A gene (157 base pairs [bp]) specific primers were 5′-CAGGGGACCTGGAAGTATTGG-3′ (forward) and 5′-TTCAGGTTGGTCACCTCACAG-3′ (reverse). Mouse *β*-actin (263 bp) primers were 5′-GAGACCTTCAACACCCCAGC-3′ (forward) and 5′-ATGTCACGCACGATTTCCC-3′ (reverse). Amplifications were carried out in 20 *μ*L reaction mixture containing 20 ng cDNA samples, and the final concentration of 0.5 *μ*mol/L of each primer pair was added in a program comprising 10 minutes at 95°C, followed by 40 cycles consisting of 95°C for 10 s, 55°C for 30 s, and 72°C for 30 s. Data was analyzed using the Bio-Rad CFX Manger. Statistical significance of differences was assessed by Student's *t*-test.

### 2.5. Immunohistochemistry

Slides were deparaffinized in xylene and rehydrated in different concentrations of ethanol (100%, 95%, 90%, 80%, and 70%), and antigen retrieval performed using a citrate buffer. Slides were blocked (37°C, 2.5 h) with 10% fetal bovine serum and then incubated with anti-ANP32A antibody (2 *μ*g/mL) for 12 hours at 4°C. IgG-purified normal rabbit serum (2 *μ*g/mL) (I5006, Sigma, SF, USA) was used as a control. After washing in phosphate buffered saline, sections were incubated with polyperoxidase-anti-rabbit IgG for 30 min at 37°C. Signals were visualized by DAB oxidation and observed by Ti-s microscope (Nikon, Japan).

## 3. Results

### 3.1. The Distribution of ANP32A in Different Areas of Human Central Nervous System

**Figure 1:**
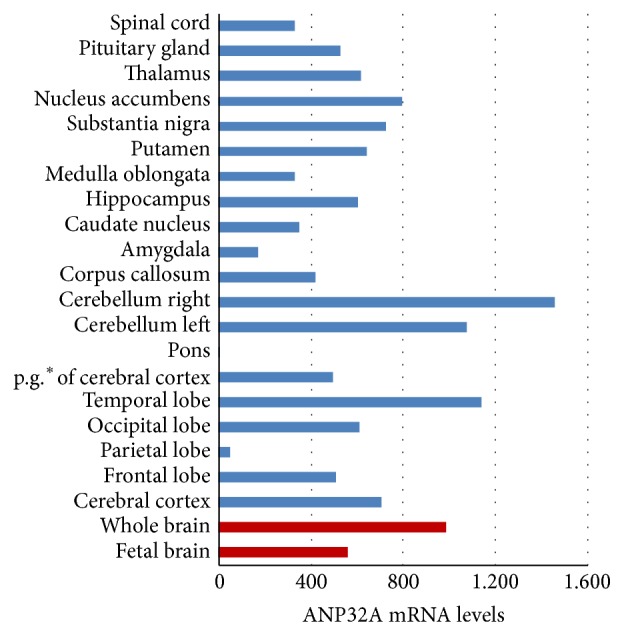
Distribution of human ANP32A transcripts in brain. Human MTE array was probed with a 750 bp human ANP32A radiolabeled probe as described under “experimental procedures.” mRNA levels were determined by densitometric scanning of autoradiographs. Whole brain and fetal brain are shown as red column, and different anatomical region is shown as blue column.

The differences of the levels of ANP32A mRNA between various areas of human brain were tested by the spot hybridized with the human MTE assay. The MTE array provides a fast way to simultaneously compare the relative abundance of ANP32A mRNA in a wide array of tissues, normalized and immobilized in separate dots, along with several controls. It was shown that brain was the organ that expressed the most abundant ANP32A, followed with heart, liver, and kidney. As shown with blue column in Figure 1, the expression levels of ANP32A mRNA were fluctuated in different areas of human brain. Among 20 different brain tissues, cerebellum right is the area with most abundant ANP32A; next is temporal lobe followed with cerebellum left, nucleus accumbens, substantia nigra, and cerebral cortex. Although ANP32A was abundant in the most brain areas, it was hardly detected in the pons. ANP32A could be slightly detected in medulla oblongata and spinal cord, a little higher than amygdala and parietal lobe.

### 3.2. ANP32A Was More Abundant in Adult Than Embryonic Brain of Both Human and Mouse

**Figure 2:**
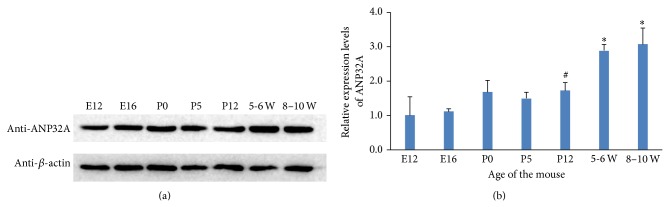
Expression of ANP32A gene in mouse developing brain. (a). Total proteins from the developing C57 BL/6 whole brains were analyzed by western blotting; (b). Total RNAs from the developing C57 BL/6 whole brains were analyzed by qPCR. E12 and E16, embryonic days 12 and 16, respectively; P0, P5, and P12, postnatal days 0, 5, and 12, respectively; 5-6 W and 8–10 W, adult brain from 5-6 weeks and 8–10 weeks old C57 BL/6. ∗: compared to E12 and E16, *P* < 0.01; #: compared to E12 and E16, *P* < 0.05.

The difference between the levels of ANP32A mRNA in adult human brain and fetal human brain was also analyzed by MTE array. Brain in embryonic stage was still the organ with most abundant ANP32A, compared with 7 important human fetal organs including heart, liver, and kidney. The level of ANP32A mRNA in the adult whole brain was about 1.5-fold that in the fetal brain (as shown with red column in Figure 1). Then the expression of ANP32A gene in a different developmental stage of C57 BL/6 brain was studied. It was shown that the expression of ANP32A was higher in adult brain than that in embryonic brain of C57 BL/6 by both western blotting and qPCR. Although it is not too significant, the rising of the levels of ANP32A protein was confirmatory during the growing of the mice in a time-dependent manner (Figure 2(a)). As for the levels of ANP32A mRNA in mouse brain analyzed by qPCR, it tended to increase after birth and was apparently raised in postnatal day 12 (P12), about 1.5-fold compared to embryonic day 12 and 16 (E12 and E16). When the mice were 5-6 weeks to 8–10 weeks old, the levels of ANP32A mRNA in the brain were increased significantly, reaching about 3-fold than that in E12 and E16 (Figure 2(b)). There was no significant difference in the interior-group.

### 3.3. The Expression Characteristics of ANP32A in the Developing Mouse Cerebral Cortex

**Figure 3:**
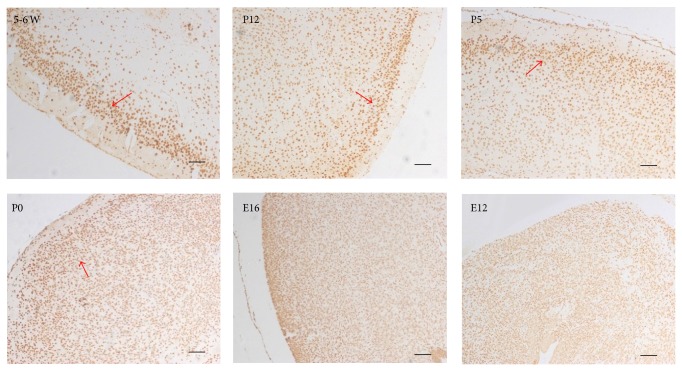
Immunohistochemical study of ANP32A in mouse cerebral cortex. ANP32A signal was visualized with DAB in C57 BL/6 brain. E12 and E16, embryonic days 12 and 16, respectively; P0, P5, and P12, postnatal days 0, 5, and 12, respectively; 5-6 W, adult brain from 5-6 weeks old C57 BL/6. Scale bar = 200 *μ*m. Granule cells were marked by red arrow.

Compared with the embryonic mouse, ANP32A protein was expressed along with the differential cerebral cortex's layer, more and more significantly, after birth. The changes of morphology of the positive stained cells were also coincidental to the development of the neuron in the nervous system. In the embryonic period, the layer of cerebral cortex is not clear. All positive staining cells are small and numerous, and the expression of ANP32A seemed to be nothing special. When the mice were birthed and growing, the positive staining cells were bigger and more strongly stained in external granular layer of the cerebral cortex (Figure 3).

In detail, from the day of birth (P0), the molecular layer of cerebral cortex appeared, and the staining of the ANP32A in the molecular layer was initially decreased than other partitions of cerebral cortex. On the P5 and P12, the positive stain cells were stratification and conversely in the molecular layer and external granular layer. The difference of staining between the molecular layer and external granular layer in 5-6 weeks old mouse brain was much more significant. The expression of ANP32A was fairly abundant in the external granular layer, localized in the nucleus of the neurons, while being apparently lower in molecular layer.

### 3.4. The Cellular Localization of ANP32A in the Granule Cells and the Purkinje Cells in the Developing Mouse Cerebellum

The distribution of ANP32A changed with the migration of external granule cells in the developing mouse cerebellum. In the embryonic period, the ANP32A was expressed moderately in the nucleus of the internal granule cells and strongly in the external granule cells. In the postnatal day 12, the signals in internal granular layer became stronger, while in the 5-6 W mouse brain the ANP32A's expression in the internal granule cells became much stronger which may be attributed in large part to migration of the external granule cells to internal granular layer.

**Figure 4:**
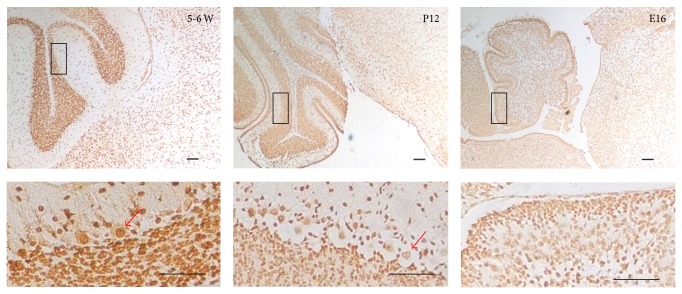
Immunohistochemical study of ANP32A in mouse cerebellum. ANP32A signal was visualized with DAB in C57 BL/6 brain. E16, embryonic day 16; P12, postnatal day 12; 5-6 W, adult brain from 5-6 weeks old C57 BL/6. In each image, the upper rectangle inset is magnified and displayed in the corresponding lower image. Purkinje cells were marked by red arrow. Scale size = 100 *μ*m.

The amount and cellular localization of the expression of ANP32A in Purkinje cells seemed to be associated with the mice age and the position in the adult mouse gyrus cerebelli. In the cerebellum P12, ANP32A was expressed moderately in only the nucleus of the Purkinje cells, which scattered evenly between the molecular layer and the granular layer. In the cerebellum 5-6W, it was absorbing that ANP32A was localized in both the nucleus and the cytoplasm, as well as dendrites arborization of Purkinje cells (Figure 4). The signals became weaker in the Purkinje cells in the root of the gyrus cerebelli. On the other hand, the signals of ANP32A became stronger in the Purkinje cells in the head of the gyrus cerebelli. It is implied that ANP32A may associate with the differentiation and function of Purkinje cells.

## 4. Discussion

ANP32 family members had been thought all functionally redundant in vivo. Because loss-of-function mutants for ANP32 family members include two independently targeted ANP32A-deficient mice [2, 28], an ANP32E-deficient mouse [2, 29] was viable and fertile. No obvious abnormalities in any of the major organ systems, including the nervous system, could be observed. However, a recent prominent finding is that mice carrying ANP32B mutations are sensitized to loss of ANP32A. The study revealed previously hidden roles for ANP32A in mouse development by compound mutants lacking ANP32A, ANP32B, and/or ANP32E [2]. Since ANP32 family members are not completely redundant in mammals, it is reasonable to presume that they may engage in regulating mechanism in the mammal development in a hierarchical and successive manner.

In view of a broad array of physiological activities [15–20] and roles in nervous system disease [21–24] of ANP32A, the description of the expression and distributions characteristics of ANP32A in the developing brain would provide strong clues for the regulating mechanism of ANP32A in the nervous system development and disease. So in this study, the distribution of ANP32A in different areas of human central nervous system, as well as the expression levels in the fetal and adult brain, was analyzed by a human multiple tissue expression array. The cellular localization and expression levels fluctuation of ANP32A were detected in the developing mouse brain. Our results indicated that ANP32A may play an important role in human nervous system development and differentiation.

MTE array showed that the expression of ANP32A was higher in the human adult brain than the fetal brain. The similar evidences have been collected by the analysis of ANP32A abundance in a series of different time point mouse brain from embryonic stage to adult stage. Both ANP32A mRNA and proteins were elevated in a time-dependent manner in the developing mouse brain. Mutai et al. had reported that expression of PAL31/ANP32B mRNA and protein in the rat brain was high during the fetal period and decreased after birth [30]. Collecting these evidences, it implied that ANP32A and ANP32B are not completely functionally redundant along with observation of compound mutants lacking ANP32A and/or ANP32B [2]. ANP32B may mainly function in fetal period of mammal, while ANP32A may primarily play roles in adult brain.

In the embryonic mouse brain, all positive staining cells were distributed homogeneously, small and numerous. The expression of ANP32A seemed to be nothing special except that the staining in the external granule cells of cerebellum was a little stronger. However, in postnatal day 12, the signals in internal granular layer became stronger. ANP32A was significantly abundant in the granular layer of the cerebellum, and the cerebral cortex when the mice were 5-6 weeks old, as well as in the Purkinje cells of the cerebellum. It may be attributed in large part of migration of the external granule cells to internal granular layer [31].

The amount and cellular localization of the expression of ANP32A in Purkinje cells seemed to be associated with the mice age and the position. In the cerebellum P12, ANP32A was expressed moderately in the nucleus of the Purkinje cells, similar to some ANP32 proteins [25, 28], which scattered evenly between the molecular layer and the granular layer. However, in the cerebellum 5-6 W, it was observed that ANP32A was localized in both the nucleus and the cytoplasm, as well as dendrites arborization of Purkinje cells. And the abundance of ANP32A in the Purkinje cells was varied according to the site of gyrus cerebella, more strongly stained in the head and less stained in the root of the adult mouse gyrus cerebella by immunohistochemistry. It indicated that ANP32A may associate with the differentiation and function of Purkinje cells [32–34].

The distribution of ANP32A in the different adult brain areas was dramatically diverse. Strongly stained nuclei were observed in the external granular layer of cerebral cortex and the granule cells in the cerebellum. MTE array showed that ANP32A was abundant in the human nervous system with high expression in cerebellum, temporal lobe, nucleus accumbens, substantia nigra, and cerebral cortex.

The cerebellum is a region of the brain that plays an important role in motor control. It may also be involved in some cognitive functions such as attention and language, and in regulating fear and pleasure responses. Its movement-related functions are the most solidly established. Learning how to ride a bicycle is an example of a type of neural plasticity that may take place largely within the cerebellum [31]. The cerebral cortex plays a key role in memory, attention, perceptual awareness, thought, language, and consciousness. The temporal lobe is one of the four major lobes of the cerebral cortex in the brain of mammals. It is involved in the retention of visual memories, processing sensory input, and comprehending language [35]. Research has indicated the nucleus accumbens has an important role in pleasure including laughter, reward, and reinforcement learning [36]. The substantia nigra plays an important role in reward, addiction, and movement. Altogether, it implied that ANP32A may have important functions in neural plasticity for essential acquired ability and participate in advancing nervous activity, such as language, emotion, learning, and memory.

On the other hand, ANP32A was lowly expressed in pons, medulla oblongata, and spinal cord. These areas of nervous system function primarily in vital activity, such as control sleep, respiration, swallowing, and motor organization. ANP32A should have tiny effects on these functional areas. In this context, it is not surprising that loss-of-function mutants for ANP32A are not fatal.

In conclusion, ANP32A was abundant in the central nervous system. The expression of ANP32A in the developing brain was raised in a time-dependent manner. And the distribution of ANP32A changed dramatically in different brain areas and layer of cerebellum or cerebral cortex, which implied the roles of ANP32A involved in differentiation and specific functional regulation of neurons. Potential mechanisms of ANP32A in the development and differentiation of nervous system may be involved in neuritogenesis modulating, apoptosis regulating, and transcription control, according to the protein localization in and out of the neurons [22, 37, 38].
